# Blood from a stone: funding hypertension prevention, treatment, and care in low- and middle-income countries

**DOI:** 10.1038/s41371-021-00583-8

**Published:** 2021-07-30

**Authors:** Jennifer Cohn, Deliana Kostova, Andrew E. Moran, Laura K. Cobb, Anupam Khungar Pathni, Dawit Bisrat

**Affiliations:** 1 Resolve to Save Lives, New York, NY United States; 2 Division of Infectious Diseases, University of Pennsylvania School of Medicine, Philadelphia, PA United States; 3 Division of Global Health Protection, Center for Global Health, Centers for Disease Control and Prevention, Atlanta, GA United States; 4 Division of General Medicine, Department of Medicine, Columbia University, New York, NY United States; 5 Resolve to Save Lives, New Delhi, India; 6 Resolve to Save Lives, Addis Ababa, Ethiopia

**Keywords:** Hypertension, Health care

## Introduction

Cardiovascular disease (CVD) is the leading cause of death worldwide, causing ~31% of all deaths [1]. The economic costs of premature death and disability from CVD are enormous: between 2011 and 2025, the estimated financial loss due to CVD in LMICs was $3.7 trillion, representing 2% of GDP of LMICs on average [2].

Hypertension, the principal cause of CVD mortality, is common, with an estimated 1.4 billion people living with hypertension globally. Rates of uncontrolled hypertension, defined by blood pressure systolic ≥140 mmHg or diastolic ≥90 mmHg, are high across low- and middle-income countries (LMICs), with LMICs suffering from disproportionate rates of premature mortality [3]. Hypertension is both a preventable and modifiable risk factor for CVD, and low-cost, effective treatments reduce risks of morbidity or mortality [4]. Unfortunately, despite the scale of disease burden from hypertension, and the availability of prevention and treatment solutions, high blood pressure is controlled in <10% of people living with hypertension across LMICs [5].

Hypertension programs in LMICs are often underfunded. While hypertension leads to significantly more deaths in LMICs than other conditions, ranking of funding priorities does not match the disease burden [6]. Since 2000, external funding for noncommunicable diseases (NCDs) in LMICs has stagnated at under 2% of total development assistance for health (DAH) despite NCDs accounting for the majority of the disease burden. Further, while total health spending has increased worldwide, the contribution and growth of patient out-of-pocket health spending has outstripped other sources in lower-income countries [7]. Currently, individuals and households shoulder significant health care costs, including for hypertension care—an inequitable situation that results in avoidance of preventive or chronic care due to inability to pay, poor patient outcomes, and increased vulnerability to catastrophic health spending when acute and severe outcomes strike [8, 9]. Countries waking up to the consequences of hypertension must increase overall public investments in health to achieve improved hypertension control at the population level while limiting the financial impact on individuals and families. Fortunately, by optimizing program design, hypertension control can be achieved efficiently and cost-effectively, resulting in affordable and sustainable programs that yield population health benefits.

## An ounce of prevention

Sodium intake may be the single most important modifiable factor for preventing elevated blood pressure in LMICs [10]. Evidence-based interventions can successfully decrease sodium intake at the population level [11]. The WHO has labeled several as “best buys,” estimating that $1 USD spent on these cost-effective interventions corresponds to $13 USD return (Table 1) [12]. Among these, regulations to reduce sodium in packaged foods, front of pack labeling to help inform consumers of products that are high in certain nutrients such as sodium, and mass media campaigns cost from $7 to 42 international dollars (a hypothetical unit with the purchasing power of a USD across currencies)/DALY averted across LMICs [13]. In comparison, the most cost-effective policy interventions for tobacco use reduction such as taxation are around $10 USD/DALY averted and patient-level interventions to manage hypertension in LMICs are just below $1000 USD/DALY averted [14, 15].

**Table 1:** Cost-effectiveness, impact, and costs of sodium reduction interventions, adapted from WHO “best buys” and other recommended interventions for the prevention and control of noncommunicable diseases [12].

Intervention	Low- and lower-middle-income countries			Upper-middle and high-income countries		
	Average cost-effectiveness (I$/DALY averted)	Health impact per year (DALY averted per one million people)	Economic costs of implementation per year (I$ in millions per one million people)	Average cost-effectiveness (I$/DALY averted)	Health impact per year (DALY averted per one million people)	Economic costs of implementation per year (I$ in millions per one million people)
Engage industry in voluntary reformulations	<100	3698	<0.01	<100	3315	<0.01
Establish a supportive environment in public institutions	<100	1085	<0.01	<100	1164	<0.01
Behavior change and mass media	<100	760	0.03	<100	819	0.02
Implement front of pack labeling	<100	2200	0.01	<100	2011	0.02

These interventions can be not only cost-effective, but also low-cost from the government perspective. A study from Argentina demonstrated that setting salt limits on bread and conducting mass media campaigns directed at lowering salt intake were both cost-effective at $50 USD/DALY averted and $179 USD/DALY averted, respectively, and affordable, with annual costs to the government for implementing each intervention in all of Buenos Aires of just $28,690 and $207,974 USD, respectively [16].

### Clinical hypertension diagnosis, care, and treatment efficiencies

**Fig. 1:**
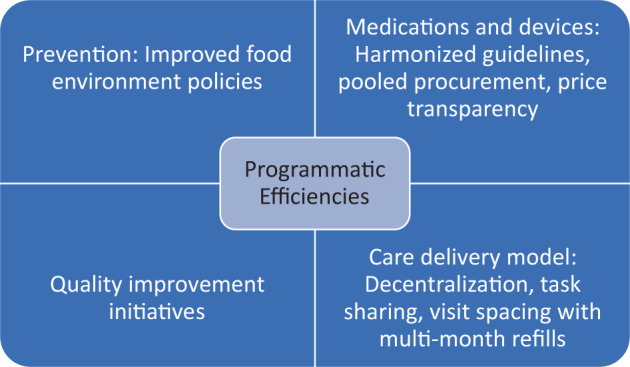
Areas for realizing efficiencies in hypertension programming: Key areas for cost efficiencies and cost savings in cardiovascular disease prevention and treatment, focusing on medications and devices, quality improvement, and care delivery models.

In addition to ramping up prevention, there are significant efficiencies to be gained in care and treatment of hypertension. Diagnosis and treatment of hypertension have been shown to be cost-effective in LMICs [15]. To accelerate national scale-up of hypertension control programs, significant additional efficiencies must be realized in medication and health system costs (Fig. 1).

### Products: medications and devices

There is significant room for price reductions for both antihypertensive medicines and blood pressure measurement devices. In many LMICs, the public sector price for generic versions of common antihypertensives is higher, often two to fourteen times more expensive than international reference prices and more expensive than public procurement prices obtained in upper-middle-income countries [17]. Obtaining prices closer to international reference prices would result in considerable reductions in countries’ medication expenditures. Tools to reduce prices include harmonizing treatment protocols, pooled procurement, increased transparency, and use of low-cost generic fixed-dose combinations (FDCs) [18].

Normative guidance supporting the use of blood pressure monitoring devices that meet WHO-recommended standards and the use of drug- and dose-specific hypertension treatment protocols enables countries to adopt and harmonize guidelines, increasing volumes for requisite medicines and devices. In HIV, guideline harmonization has led to a 70–80% price reductions for first-line medicines [19]. In 2018, the WHO released its HEARTS package with a number of treatment algorithm options for hypertension treatment and CVD prevention [20]. Future WHO treatment guidelines offer the opportunity to distill this guidance further, resulting in global standard treatment protocols that consolidate the market for hypertension medications.

Beyond focusing on improving the affordability of a limited number of preferred products, a number of market-based approaches may help to reduce medication and blood pressure device prices. Given the high prevalence of hypertension in LMICs, the scale of need is large, affording significant purchasing leverage. Pooling volumes through centralized procurement within one country or regionally between countries, as for example the PAHO strategic fund does for the PAHO region, can lead to price decreases of up to 90% across a range of medications, including cardiovascular treatments [21].

Increasing price transparency through collecting and disseminating market information may also facilitate price reductions for essential medical products. Price transparency allows buyers to compare among various suppliers in a market, facilitating competition and possibly accelerating price decreases [22]. However, this approach must reflect other market circumstances such as the current or potential growth, to incentivize manufacturers to stay in the market and offer competitive pricing [23]. Although there are resources for HIV, TB, and malaria medications, fewer platforms exist to provide a transparent exchange of information on hypertension medication prices and availability.

Finally, using generic FDC of hypertension medications as opposed to combinations of single agent pills can reduce medication costs due to manufacturing or other efficiencies. A study of antihypertensives sold in the private sector in India demonstrated that the price of FDCs could be lower than their component single agent pills [24]. Similar price trends have been seen in HIV [25].

### Health system efficiencies

Hypertension care in many LMICs is centralized, with patients being seen at higher-level facilities and requiring frequent follow-up appointments [26]. This model of care may not be sustainable for the health system or patient. Given health system constraints in LMICs, there are not enough specialists, or doctors, to address the anticipated demand for hypertension care [27]. Task shifting—enabling less-specialized health workers with training, support, and where appropriate, certification, to diagnose, initiate, and maintain treatment—can help use limited resources more efficiently [28]. Evidence from other disease programs shows task shifting to be safe and effective and evidence is growing to support its use in hypertension [29]. Evidence from HIV programs, where task shifting is common, has demonstrated up to 66% reduction in government expenditures on HIV follow-up care by task shifting to nurses or pharmacists [30]. A model based on Indian data suggests task shifting to nurses can enable India to meet the potential demand for hypertension care without expanding the health workforce [31].

The use of decentralized and differentiated service delivery (DSD) models can help save costs both from the health system and patient perspective. Decentralization refers to provision of care or specific interventions at lower-level primary health care facilities. Decentralization alone significantly reduces out-of-pocket costs for chronic diseases in LMICs [32]. DSD incorporates both task sharing and decentralization into a comprehensive care model that also tailors care intensity to meet the clinical needs of the patient. HIV is a disease, which mirrors hypertension in its service delivery needs in that it is a chronic, lifelong disease that is potentially deadly if not treated, but can be managed with safe and effective daily oral medicines at the primary care level. In HIV, DSD is extensively used and supports the implementation of less-intensive models of care for well-controlled patients. Changes in follow-up frequency through use of extended drug refills of 3–6 months can significantly reduce the financial and time burden for both patients and providers, with data from HIV indicating price savings of up to 66% for patients and 10–15% for health systems [33].

Efficiencies may be realized by ensuring quality care for those on treatment. Improvements in health care quality are an important condition for accomplishing the objectives of increased access to care [34]. Hypertension programs can lose limited resources when patients who are diagnosed and enrolled in the hypertension program are lost to follow-up or do not attain hypertension control. Quality improvement starts with a health information system that tracks individual patients and feeds back program performance indicators. Informed by health information system data, implementation of decentralization, DSD, and other quality-improvement measures can improve treatment adherence and retention in care in LMICs. The use of single-pill, FDC treatments has also been demonstrated to result in higher levels of adherence and control as compared to the same combinations using separate agent pills, and can furthermore improve supply chain efficiencies [35]. Quality of hypertension care in resource-limited environments can also be strengthened through training of primary care providers in standardized treatment and prevention approaches, as outlined in the World Health Organization HEARTS technical package. Standardized protocols for diagnosis and treatment of hypertension in primary care settings can support scalability and population outreach.

In health programs with limited resources, approaches for short-term cost-savings must be weighed against potential negative impact on health outcomes and loss of long-term efficiencies. For example, as countries face a growing demand for NCD services, LMICs may employ user fees that patients to pay for services out-of-pocket to meet costs. These approaches need to be carefully calibrated to balance the need for cost containment with the need for optimal health care. In LMICs, where out-of-pocket spending constitutes a major share of health expenditure, user fees are associated with worse health outcomes and may lead to catastrophic costs for individuals [8]. Other approaches may help to close the gap between rising health needs and available resources, including optimal reallocations of government expenditure devoted to health and implementation of progressive taxes that help to increase the funding base. For example, implementation of taxes on tobacco and alcohol in the Philippines nearly doubled the country’s internal health budget, and the added resources helped increase access to social insurance programs and alleviate the burden on households [36]. For LMICs, where the shortfall between health needs and resources is expected to persist over time, DAH will continue to play a significant role [37]. In this case, a closer realignment of DAH to disease burden patterns in LMICs may help better guide future resource allocation [38]. In the short term, DAH funding for NCD programs in LMICs can help fill health sector gaps in NCD care.

## Conclusion

Scaling up effective hypertension programs is critical for controlling the growing burden of CVD, but implementing and sustaining these programs at a national scale will require additional investments by countries. Despite the clear evidence that hypertension prevention and treatment is cost-effective and saves lives, current funding constraints mean that presently programs do not provide adequate coverage to meet population health needs. However, programs can be made more efficient and less expensive per individual attaining control—potentially significantly less expensive—and countries and other health care payers can stretch their money by exploiting the efficiencies reviewed in this paper and ensure maximum benefit for the populations they care for. In many countries, robust hypertension control programs do not yet exist, and program start-up may require up-front financial investment, aggressive policy change, and adoption of new models of care. The medium- and long-term payoff from this up-front investment will lead to more affordable programs while expanding coverage, improving control, and reducing cardiovascular morbidity and mortality.

## Disclaimer

The findings and conclusions in this report are those of the authors and do not necessarily represent the official position of the Centers for Disease Control and Prevention.

## References

[1] Roth GA, Mensah GA, Johnson CO, Addolorato G, Ammirati E, Baddour LM (2020). Global burden of cardiovascular diseases and risk factors, 1990–2019: update from the GBD 2019 Study. J Am Coll Cardiol.

[2] World Health Organization and World Economic Forum. From burden to “best buys”: reducing the economic impact of non-communicable diseases in low and middle-income countries. Geneva: World Economic Forum; 2011.

[3] NCD Risk Factor Collaboration (NCD-RisC (2017). Worldwide trends in blood pressure from 1975 to 2015: a pooled analysis of 1479 population-based measurement studies with 19.1 million participants. Lancet.

[4] Ettehad D, Emdin CA, Kiran A, Anderson S, Callender T, Emberson J (2016). Blood pressure lowering for prevention of cardiovascular disease and death: a systematic review and meta-analysis. Lancet.

[5] Geldsetzer P, Manne-Goehler J, Marcus M, Ebert C, Zhumadilov Z, Wesseh CS (2019). The state of hypertension care in 44 low-income and middle-income countries: a cross-sectional study of nationally representative individual-level data from 1·1 million adults. Lancet.

[6] Bollyky TJ, Templin T, Cohen M, Dieleman JL (2017). Lower-income countries that face the most rapid shift in noncommunicable disease burden are also the least prepared. Health Aff.

[7] Global Burden of Disease Health Financing Collaborator Network. (2019). Past, present, and future of global health financing: a review of development assistance, government, out-of-pocket, and other private spending on health for 195 countries, 1995–2050. Lancet.

[8] Qin VM, Hone T, Millett C, Moreno-Serra R, McPake B, Atun R (2019). The impact of user charges on health outcomes in low-income and middle-income countries: a systematic review. BMJ Glob Health.

[9] Huffman MD, Rao KD, Pichon-Riviere A, Zhao D, Harikrishnan S, Ramaiya K (2011). A cross-sectional study of the microeconomic impact of cardiovascular disease hospitalization in four low- and middle-income countries. PLoS ONE.

[10] Kontis V, Cobb LK, Mathers CD, Frieden TR, Ezzati M, Danaei G (2019). Three public health interventions could save 94 million lives in 25 years. Circulation.

[11] Ide N, Ajenikoko A, Steele L, Cohn J, J. Curtis C, Frieden TR (2020). Priority actions to advance population sodium reduction. Nutrients.

[12] World Health Organization. WHO Global NCD Action Plan 2013–20. Appendix 3. Available from: https://www.who.int/ncds/governance/technical_annex.pdf. Accessed 22 Feb 2021.

[13] Murray CJ, Lauer JA, Hutubessy RC, Niessen L, Tomijima N, Rodgers A (2003). Effectiveness and costs of interventions to lower systolic blood pressure and cholesterol: a global and regional analysis on reduction of cardiovascular-disease risk. Lancet.

[14] In: Prabhakaran D, Anand S, Gaziano TA, editors. Cardiovascular, Respiratory, and Related Disorders. 3rd ed. Washington (DC): The International Bank for Reconstruction and Development/The World Bank; 2017. 30212054

[15] Kostova D, Spencer G, Moran AE, Cobb LK, Husain MJ, Datta BP (2020). The cost- effectiveness of hypertension management in low-income and middle-income countries: a review. BMJ Glob Health.

[16] Rubinstein A, García Martí S, Souto A, Ferrante D, Augustovski F (2009). Generalized cost-effectiveness analysis of a package of interventions to reduce cardiovascular disease in Buenos Aires, Argentina. Cost Eff Resour Alloc.

[17] Husain MJ, Datta BK, Kostova D, Joseph KT, Asma S, Richter P (2020). Access to cardiovascular disease and hypertension medicines in developing countries: an analysis of essential medicine lists, price, availability, and affordability. J Am Heart Assoc.

[18] USAID. Healthy markets for global health: a market shaping primer. 2018. https://www.usaid.gov/sites/default/files/documents/1864/healthymarkets_primer_updated_2018.pdf.

[19] Waning B, Kyle M, Diedrichsen E, Soucy L, Hochstadt J, Barnighausen T, et al. Intervening in global markets to improve access to HIV/AIDS treatment: an analysis of international policies and the dynamics of global antiretroviral medicines markets. Global Health. 2010;6:9. 10.1186/1744-8603-6-9. 10.1186/1744-8603-6-9 PMC2883977 20500827

[20] World Health Organization. HEARTS technical package. Available from: https://www.who.int/cardiovascular_diseases/hearts/en/. Accessed 22 Feb 2021.

[21] Kim SungWook, Skordis-Worrall Jolene (2017). Can voluntary pooled procurement reduce the price of antiretroviral drugs? A case study of Efavirenz. Health Policy Plan.

[22] Austin AD, Gravelle J. Does price transparency improve market efficiency? Implications of empirical evidence in other markets for the health sector. CRS Report for Congress, RL34101. Washington, D.C., U.S: Congressional Research Service; 2007.

[23] Kyle MK, Ridley DB (2007). Would greater transparency and uniformity of health care prices benefit poor patients?. Health Aff.

[24] Negi S, Neupane D, Sahoo SK, Mahajan T, Swaroop K, Moran AE (2021). Prices of combination medicines and single-molecule antihypertensive medicines in India’s private health care sector. J Clin Hypertens.

[25] Medecins sans Frontiers. Untangling the web: HIV medicine pricing and access issues. 2020. Available from: https://msfaccess.org/sites/default/files/2020-11/HIV_Brief_Untangling-the-Web_2020.pdf. Accessed 1 May 2021.

[26] Vedanthan R, Bernabe-Ortiz A, Herasme OI, Joshi R, Lopez-Jaramillo P, Thrift A (2017). Innovative approaches to hypertension control in low- and middle-income countries. Cardiol Clin.

[27] Stenberg K, Hanssen O, Edejer T, Bertram M, Brindley C, Meshreky A (2017). Financing transformative health systems towards achievement of the health Sustainable Development Goals: a model for projected resource needs in 67 low-income and middle-income countries. Lancet Glob Health.

[28] Chen L, Evans T, Anand S, Boufford JI, Brown H, Chowdhury M (2004). Human resources for health: overcoming the crisis. Lancet.

[29] Anand TN, Joseph LM, Geetha AV, Prabhakaran D, Jeemon P (2019). Task sharing with non-physician health-care workers for management of blood pressure in low-income and middle-income countries: a systematic review and meta-analysis. Lancet Glob Health.

[30] Mdege ND, Chindove S, Ali S (2013). The effectiveness and cost implications of task-shifting in the delivery of antiretroviral therapy to HIV-infected patients: a systematic review. Health Policy Plan.

[31] Marklund M, Cherukupalli R, Pathak P, Neupane D, Krishna A, Wu JH (2020). Abstract P182: workforce reforms and task sharing to improve hypertension treatment coverage in India. Hypertension.

[32] Cobos Muñoz D, Merino Amador P, Monzon Llamas L, Martinez Hernandez D, Santos Sancho JM (2017). Decentralization of health systems in low and middle income countries: a systematic review. Int J Public Health.

[33] Prust ML, Banda CK, Nyirenda R, Chimbwandira F, Kalua T, Jahn A (2017). Multi-month prescriptions, fast-track refills, and community ART groups: results from a process evaluation in Malawi on using differentiated models of care to achieve national HIV treatment goals. J Int AIDS Soc.

[34] Kruk ME, Gage AD, Joseph NT, Danaei G, Garcia-Saiso S, Salomon JA (2018). Mortality due to low-quality health systems in the universal health coverage era: a systematic analysis of amenable deaths in 137 countries. Lancet.

[35] Benjamin IJ, Kreutz R, Olsen MH, Schutte AE, Lopez-Jaramillo P, Frieden T (2019). Fixed-dose combination antihypertensive medications. Lancet.

[36] World Health Organization. Available from: https://www.who.int/features/2015/ncd-philippines/en/#:~:text=Within%20two%20years%20of%20passing,are%20earmarked%20for%20specific%20programmes.&text=The%20remaining%2085%25%20goes%20to,and%20train%20doctors%20and%20nurses. Accessed 1 Apr 2021.

[37] Global Burden of Disease Health Financing Collaborator Network. (2019). Past, present, and future of global health financing: a review of development assistance, government, out-of-pocket, and other private spending on health for 195 countries, 1995–2050. Lancet.

[38] Bollyky TJ, Templin T, Cohen M, Dieleman JL (2017). Lower-income countries that face the most rapid shift in noncommunicable disease burden are also the least prepared. Health Aff.

